# Delayed Presentation of Congenital Diaphragmatic Hernia with Acute Respiratory Distress: Challenges in Diagnosis and Management

**DOI:** 10.1155/2020/6109487

**Published:** 2020-07-26

**Authors:** Kam Lun Hon, Ronald C. M. Fung, Alexander K. C. Leung

**Affiliations:** ^1^Department of Pediatrics, Prince of Wales Hospital, The Chinese University of Hong Kong and Department of Pediatrics and Adolescent Medicine, The Hong Kong Children's Hospital, Hong Kong; ^2^Department of Pediatrics and Adolescent Medicine, The Hong Kong Children's Hospital, Hong Kong; ^3^Department of Pediatrics, The University of Calgary, The Alberta Children's Hospital, Calgary, Alberta, Canada

## Abstract

Delayed presentation of congenital diaphragmatic hernia (CDH) with acute respiratory distress beyond the newborn period may poise challenges in diagnosis and management. We report a 3-month-old infant who presented with acute-onset respiratory distress and left congenital diaphragmatic hernia that was relieved with thoracoscopic repair. CDH must be differentiated from pneumothorax or pulmonary cyst. Erroneous diagnosis and treatment with thoracocentesis could be disastrous. Pediatricians and surgeons must be aware of this condition to allow early diagnosis and expeditious management. Subcutaneous emphysema should not be misdiagnosed as pneumothorax and management is expectant.

## 1. Introduction

Infants with congenital diaphragmatic hernia (CDH) most often present with respiratory distress in the immediate neonatal period. Delayed presentation of CDH with respiratory distress beyond the newborn period is unusual and may poise challenges in diagnosis and management. We report a 3-month-old girl with CDH who remained asymptomatic until 3 months of age when she presented with acute respiratory distress.

## 2. Case

**Figure 1:**
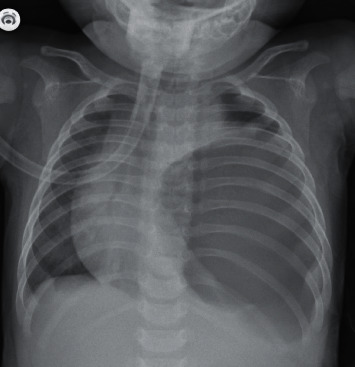
Left congenital diaphragmatic hernia with mediastinal shift to the right.

A 3-month-old girl presented to the emergency department with irritability, dyspnea, and feed refusal for a day. She was born full term and had unremarkable antenatal and perinatal course. Physical examination revealed a crying baby with cyanosis and reduced breath sounds at the left lung. Her respiratory rate was 60/min and heart rate was 160/min. The chest was barrel-shaped and the abdomen was scaphoid-like. The apex of the heart was displaced to the right. The oxygen saturation was only 44% while in ambient air, which was then normalized with 10 L/min oxygen via a face mask. Chest X-ray showed a huge gastric bubble occupying more than two-thirds of left hemithorax, causing mediastinal shift to the right side, and leaving a very small left lung field (Figure 1).

With face mask oxygenation, saturations above 95% were maintained. This is important so that the lesion was correctly diagnosed with CXR without mistreating it as tension pneumothorax by thoracostomy and chest tube insertion.

An orogastric tube was inserted. Respiratory distress was improved after aspirating 130 ml clear fluid and 100 ml air from the stomach. Thoracoscopy identified a 5 cm × 2 cm defect at the posteriolateral part of the left diaphragm, leading to herniation of the spleen, stomach, transverse colon, and greater omentum. The herniation was completely reduced, and the defect was repaired with sutures. A chest drain was inserted to allow drainage after thoracoscopy.

There was no pulmonary hypoplasia as evident by good oxygen saturations following operation.

**Figure 2:**
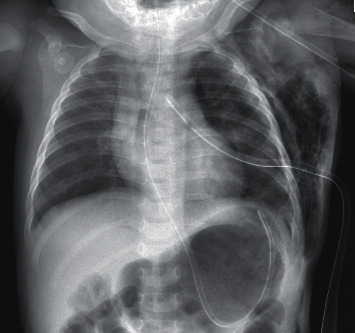
Extensive subcutaneous emphysema over the left chest.

However, significant surgical emphysema was observed on the next day (Figure 2), most likely due to a leak from the lung parenchyma. The girl was otherwise stable without any respiratory distress. Serial chest radiographs revealed resolving emphysema. She had no associated congenital anomalies.

## 3. Discussion

CDH is a development defect of the diaphragm which allows herniation of the abdominal viscera into the chest cavity [1, 2]. Bochdalek hernia, also known as posterolateral diaphragmatic hernia, is the most common form of CDH, accounting for more than 95% of cases [1, 2]. The majority of Bochdalek hernias (80–85%) occur on the left side of the diaphragm. CDH can often be diagnosed before birth by routine prenatal ultrasound screening usually around 24 weeks of gestation. Infants born with diaphragmatic hernia often experience respiratory distress due to both pulmonary hypoplasia and pulmonary hypertension. Pulmonary hypoplasia is related to the presence of abdominal organs in the chest cavity which causes the lungs to be severely undersized, especially on the side of the hernia.

Clinical manifestations, depending upon the side of CDH, include abnormal chest movements, difficulty breathing, cyanosis, absent breath sounds on the affected side of the chest, bowel sounds in the chest, and a scaphoid abdomen. Also, CDH can occur as either an isolated or complex anomaly, including genetic or chromosomal syndromes [3]. Pulmonary hypertension is due to a restriction of blood flow through the hypoplastic lung. Hypoxia and acidosis further increase the risk of pulmonary hypertension by inducing vasoconstriction.

In the majority of cases, infants with CDH present with respiratory distress at birth or shortly thereafter. Late presentation of CDH was estimated to occur in less than 20% of all CDH cases, but 80% of these delayed cases present with acute symptoms.

In one study, 68% had a prenatal diagnosis [4]. Survival rates were significantly better in the postnatally diagnosed group, 83% vs 65%. Prenatally diagnosed CDH is associated with larger defect sizes compared to those with a postnatal diagnosis and consequently have higher morbidity and mortality [3–5]. Right-sided CDH is more often missed at prenatal ultrasound.

Our patient presented with life-threatening respiratory distress and hypoxia at 3 months of age. Radiograph showed left-sided CDH, with mediastinal shift to the right. Immediate gastric decompression followed by reduction of the abdominal viscera into the abdominal cavity and thoracoscopic repair of the diaphragmatic defect relieved the respiratory distress and was lifesaving. CDH may be erroneously diagnosed as pneumothorax or pulmonary cyst. Treatment with thoracostomy may lead to rupture of the stomach with a disastrous outcome.

CDH is a life-threatening condition in infants and a major cause of death unless treated appropriately [1, 2]. Outcomes of CDH are largely dependent on the severity of the defect and the appropriate timing of treatment. Bilateral CDH is associated with a high mortality rate. A small percentage of cases go unrecognized into adulthood [6]. Sudden decompensation beyond the neonatal period is uncommon and may be disastrous due to misdiagnosis. Our case alerts clinicians to the possible presentation of CDH at a later age.

Gastric decompression (nasogastric tube placement) and maintenance of a patent airway (intubation) should be performed without delay. Blood pressure support and ventilatory support are essential. Extracorporeal membrane oxygenation (ECMO) may be necessary in patients who do not respond to supportive medical interventions [7, 8]. Fluid and electrolyte imbalance and acidosis, if present, should be corrected. Surgical repair consists of reduction of the abdominal viscera into the abdominal cavity and closure of the diaphragmatic defect. Drainage of the thorax postoperatively using chest tubes is a standard procedure in thoracic surgery. However, chest tubes can induce pain and immobilization, increase risk of infection, deteriorate the ventilation capacity, and increase difficulty of postoperative management in children [9]. Subcutaneous emphysema is often a postoperative complication, which is often asymptomatic and spontaneously resolved within 3 to 7 days.

## 4. Conclusion

Delayed emergency presentation of CDH beyond the newborn period poises challenges in diagnosis and management. Outcomes of CDH are largely dependent on the severity of the defect and the appropriate timing of treatment. Correct and timely interpretation of radiographic imaging is essential.
